# The Existing State Analysis of Working Children on the Street in Sanliurfa, Turkey

**Published:** 2018-09

**Authors:** Selma KAHRAMAN, Hülya KARATAŞ

**Affiliations:** Dept. of Public Health Nursing, School of Health, Harran University, Sanliurfa, Turkey

**Keywords:** Child, Street children, Nursing, Turkey

## Abstract

**Background:**

We aimed to determine the existing state analysis of children on the street and also attending to a school. The children’s ages were between 13–18 yr, attending grades between 7–12^th^ grade.

**Methods:**

The sample group was 54,928 students in Sanliurfa, Turkey. The data were analyzed using Chi-square test (*P*<0.05).

**Results:**

82.8% of working children were male. 92.5% were elder than 12 yr old and 85.8 % had more than 4 siblings. 52.7% of the families’ income was less than 1,000 TL per month. 79.9% of the children had worked in different places before. The distribution of working places was 66.4% in an establishment, 9.5% in the fields, 7.5% in the bazaar and in the streets, 2.8% in bus terminal, 2.9% in industry, 2.5% in parks. The 46.3% of the children stated that they are working willingly. 50.7% were working 6 to 10 h a day. 64.4% were giving the money they earned to their families. 82.1% of the children stated that they liked to attend the school but 62.8% sometimes did not go to school. 20.6% went to work instead of school in those days. The 62.2% of the children answered the question; “According to you, should the children work?” with; “No”.

**Conclusion:**

The children started working at very early ages and they faced unfavorable situations. To avoid the children work in very early ages, it is suggested to make educational programs and to compose policies to resolve the problems that force the children to go to work.

## Introduction

Youth is one of the most dynamic periods of the life. In this period, the young changes dramatically and tries to cope with the problems that these changes bring. In the youth period, besides the biological and psychological changes, the individuals also face social changes such as taking responsibilities, making decisions and taking social states (1). As this period also involves changes and effects in itself, the individuals mostly make a decision to start working (2, 3).

UNICEF defined the concept of working children as children aged 18 or less, making contributions to production with or without their family, in any time of day, even they get money or not (4).

“Working children in street”, separately from working children, are defined as children working in street for the economic reasons such as helping livelihood of their families or gaining for their own expenditures, thus, spending the major parts of their times in streets, and generally returning to their home after work (1, 3, 5). According to the data of Turkish Statistical Institute (TÜİK-2012), between the 7 to 17 yr old, 893 thousand children work in Turkey. 4.7% of children work in the agricultural sector; 24.3 % in industrial sector; and 31% in service sector (6). However, although, many statistical reports regarding working children were published in the world and in Turkey, it is difficult to determine the number of children working in street exactly (7, 8).

Although working children in street are living with family, so this will reduce the risks and danger they face, both working children in street and street children face to all risks and dangers. Besides the danger in street, working children in street are also negatively affected by many aspects such as anatomic, physiological, and psychological due to working in street especially in adolescence period (9–11).

The increase observed in the number of children working and wandering in the streets leads to new concerns in Turkey and this is a big problem that must be overcome. Children working or maybe also living in street are in the first place that indigent to the specific protection precautions. Therefore, it is important to know the number of working children and to identify their states for providing care for them, orientating them in an appropriate way, and protecting them from the risky behaviors (12–14). According to the data obtained from the State Institute of Statistics (DIE), the child population in Sanliurfa is higher than the other cities in Turkey. We aimed to analyze the existing states of working children in Sanliurfa, Turkey (12–14).

## Materials and Methods

This study was carried out to identify the analysis of existing situation of the children attending the school between seventh grade and twelfth grade and also working in street in Sanliurfa, Turkey.

The research group was composed of children, (54928 students) attending between 7–12 grade of secondary schools and high schools of Provincial Directorate of National Education in Oct–Dec 2014. The collected data from 54928 students were entered into the SPSS 16 software (Chicago, IL, USA) and the number of working children on the street was found at 4.541 students. The findings were obtained from those 4541 children.

The data were collected by using questionnaire method, prepared by the researchers in the direction of the relevant literature. While applying the questionnaire, the method of face to face interview was utilized. In addition, the explanations were made for all groups of samples that administration of survey was based on voluntariness and that the answers they gave would be kept confidential. After giving information to all sample groups, they were asked to reply the questions. Filling out the questionnaire took 5–10 min and the participation rate was 84%.

While preparing the questionnaire, opinions of the specialists were taken. Then, the questionnaire was arranged in the direction of their suggestions and a pre-administration was made among 15 students. The following titles were examined in the scope of the study. Questionnaire was composed of two sections. In the first section, the demographic data belonging to the child and family took place, while in the second section there were questions regarding the status of working.

Written permissions from the institutions and the verbal consent of the participants were obtained for the study. It was explained to the entire sample that the surveys were voluntary and that their answers would remain confidential. Then they were informed about the study and asked to answer the surveys. This study was supported by Karacadağ Kalkınma Ajansı (Project No: TRC2/14/DFD/0007).

The collected data were carried out on SPSS 16. In this study, socio-demographic features of the children and families constituted the independent variable, the working status of children constituted dependent variable. As a correlation test, Chi-square test was conducted and the value of s <0.05 was accepted as significance level of statistical tests. In the assessment of data, mean, standard deviation, median, minimum and maximum values, and percentage numbers were used.

## Results

Sociodemographic features are presented in Table 1. 82.8% of the working children were male and 92.5% of them were more than age 12. 41.7% of the children were the 4^th^ child of the family or more. 62.7% had a social activity, and that 48.1% of the children did sports as social activity.

**Table 1:** Socio-Demographic Properties of Children

***Variables***	***n***	***%***
Gender		
Male	3.759	82.8
Female	659	14.5
Not stated	123	2.7
Age(yr)		
Age 12 and more	259	5.8
More than age 12	4.211	92.5
Not stated	76	1.7
Which child in order		
First	781	17.2
Second	864	19.0
Third	803	17.7
Fourth and more	1.895	41.7
Not stated	198	4. 4
Social Activity		
Yes	2.848	62.7
Non	1.403	30.8
Not stated	290	6.4
Kind of Social Activity N=3.575		
Sports	1.721	48.1
Music	599	16.8
Reading	733	20.5
Other (travel. cinema. etc.)	522	14.6

The mother of 54.6% of the children and father of 15.1% were illiterate. 19.9% of fathers did not work in any place and 52.7% of families had a monthly income of less than 1,000 TL. 79.3% of the children defined their families as an elementary family (composed of parents and children), and 41.1% of children told that the decisions were made by father only. 85.8% of the children had siblings 4 and more.

79.9% of working children in street previously worked for making money and 46.1% of them began to work in the age of less than 10 (Table 2).

**Table 2:** The features of child related to working in street

***Variable***	***n***	***%***
The case of the child related to working for previously making money		
Yes	3.628	79.9
No	604	13.3
Not stated	309	6.8
The age that the child begins to work		
Age 10 and less	2.044	46.1
More than age 10	2497	53.9
Sort of job n=3.159		
Repair - Apprenticeship	1.518	48.1
Selling handkerchief. bagel. etc.	136	4.3
Freightage in bazaar	133	4.2
Working in baker	429	13.6
Shoeshine paper gathering. shop assistant.	219	6.9
Begging	189	6.0
Car wash	140	4.4
Agricultural worker	395	12.5
Working Hour		
5 h and less	900	27.5
6 –10 h	1.657	50.7
More than 10 h	713	21.8
Working Place n=3.057		
In Park	75	2.5
In coach station	87	2.8
In Bazaar	230	7.5
In workplace. in baker	2.030	66.4
In industry	90	2.9
Infield	291	9.5
In street	230	7.5
Form of Spending Money		
I spend	1.050	29.7
I deliver to my family	2.274	64.4
Both of them	206	5.9
The case of spending the night outside due to working		
Yes	630	13.9
No	2.791	61.4
Not Stated	1.127	24.7
The case of his/her sibling working		
Yes	2.289	50.4
Not	2.001	42.0
Not Stated	341	7.6
Do you think that the children should work?		
Yes	1.329	29.3
Non	2.826	62.2
Not stated	386	8.5
What is the harm of working to the child? n=1.536		
His/her school success falls.	631	41.0
He/she does not become healthy physically and psychologically	453	29.4
He/she is inclined to accidents	255	16.8
He/she has harmful habits	197	12.8
The problems he/she experiences due to working n=3.230		
Headache	510	15.7
Backache	609	18.8
Leg pain	182	5.6
Tiredness	1.025	31.3
Sunstroke Burn. Skin injuries. contagious disease	136	4.2
All of them	768	23.7

48.1% of the children worked in repairing and apprenticeship; 50.7% of them worked between 6–10 h; and 66.4% of them worked in the work-place or in a baker.

64.4% of the working children stated that they delivered the money they gained to their families; 46.3% of children stated that they were absent from school due to health problems, 20.6% of them were due to working.

13.9% of them spent the night working outside. The siblings of 50.4% of the children were also working.

**Fig. 1:**
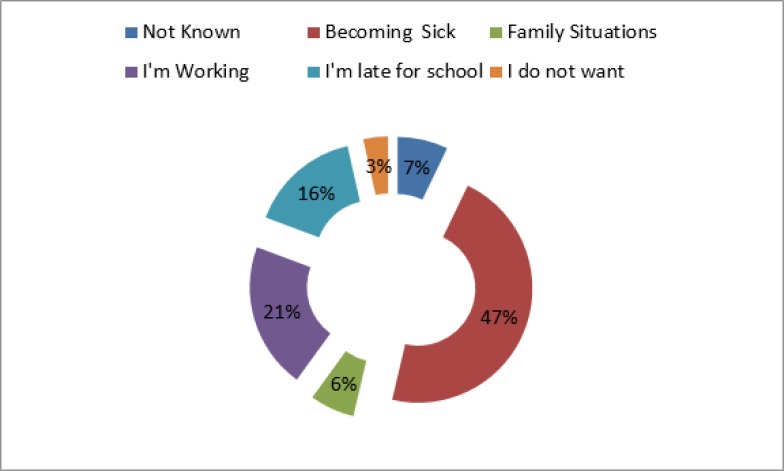
The cause of being absent from school for the children

While 29.3% of children said that children should not work, 41.0% of them expressed that working in childhood reduced school success; and that 18.8% experienced backache due to working. Overall, 14.0% of children smoked cigarette, and 4.5% used drugs (Fig. 1).

18.0% of children expressed that they exposed to violence by their families, and 14.7% were exposed to the violence due to working. 67.7% of the children exposed to violence stated that they underwent to the violence by their fathers, and 33.3% of them by the owner of workplace.

21.0% of the children spent the night outside and 37.4 % were obliged to spend the night outside since they work.

## Discussion

The phenomenon of working children in street is an economic and social problem and the most important agenda item of the world, waiting for prior solution (5). Especially in order to make a contribution to the income of family, children begin to work in marginal jobs; as a result, they interrupt their educations. Thus, as the physical, mental, spiritual, and social development are not completed yet, the various factors in working life negatively affect the health of working children (2, 8, 11, 15).

In this study, 82.8% of working children were male and almost all of them (92.5%) were more than age 12 (Table 1). In this study, the average age was 15.42. In Turkey, according to a study carried out with 188 working children in street living in Diyarbakır, Adana, and Istanbul, the average age of these children is 12 and they are mostly male (3). In Turkey, 71% of the working children are between the ages of 11–13 and that 94.1% of them are male (16). In another study, 58.1 % of the students begin to work in the ages of 10–12 and 25.7% in the ages of 7–9 (7). This study exhibits a similar profile to the results of those studies.

When regarded to the gender of the working children, mostly the boys were forced to work in this study. In Turkey, this rate is identified as 61.8% for boys, and 38.2% for girls (8). The reason for the difference in a number of the working boys in the present study is because the girls were left in the houses and they worked as house workers while boys were forced to work in street. In this study, 85.2% of children had siblings more than 4. In another study, 92.9% of the students have sibling more than 3 (12). All over Turkey, among the families, whose members are 7 or more, the rate of poor families is 45.95%. The working children mostly come from the crowded families (14). Although the average of the number of the member of the families is below 4 in Turkey, the working children are mostly from the families with 4 or 5 children and even more, so it makes us think the similar solutions.

Another reason for child labor is traditional viewpoint. This viewpoint is affected by the education, income, and other familiar features. In this study, when the educational status of the working children’s mothers was under consideration, 54.6% of them were illiterate. For fathers, although this rate was less, they also had low educational level. When regarded to the income status of family, it was identified that the monthly income of families was less than 1,000 TL. 41.1% of children told that the decisions were made by father only. This result is similar to the results of another study (17). The child, who do not want to place a strain on his family, immediately directs to have a work. From the sociocultural viewpoint, child labor is accepted as normal, and sometimes, it is also seen as necessary. In both situations, families do not have the adequate information about the negative effects of working life on children and for this reason; families continue to let their children work in street (11). Most of the families do not take into consideration the risks the children expose due to working in street. Perhaps, the most important finding of this study is that, every second child in the sample was in the state of poverty and deprivation and this situation made them [families] thought that working of child in street had positive – approving social perceptions and the child could be viewed as a buffer instrument while struggling against poverty (1, 2).

In this study, in order to identify the ratio of the working children among 7–12^th^, the state of working at the moment were found 10.2%, while the rate of the previous working was identified as 29.2%. The rate of working child in the world was specified as 10% (4). In Turkey, in 2006, while 2% of the children attending school were working in an economic activity, this rate reached 3% in 2012. According to the results of child labor survey realized in the period IV of year 2012, 5.9% of the children worked in economic jobs (18). When these rates are compared, the number of working children in Sanliurfa are considerably higher than average of Turkey and moreover, it is also higher from the average in the world. The solution studies toward child labor should primarily begin in the cities such as Sanliurfa.

In this study, 79.9% of the children previously worked to make money. When regarded to the case of working of siblings of these children, 50.4% of them worked. The working children thought that they were good samples for their brothers or sisters as they worked. Even these children thought that their families exemplified them to other children as “become like elder brother”, and motivated them to work also. In this study, 62.7% of the working children made social activity. 72.5% of them worked more than 5 hours (Table 2). Working duration is long, states that one can face with not being able to adequately satisfy the main needs such as sleeping, resting, having fun, and social relationships. Working children may experience physical, emotional, or sexual exploitation in working environment. In addition to these, the children cannot find the necessary possibilities for healthy nutrition. It is very important to completely meet the nutrition needs of the children at this age. Working children in street impose too heavy works exceeding their power, and since they spend their times out of school by working in street, they become distant from the school after a while, they do not experience the activities specific to childhood, and expose to violence in the places they work, and undergo to the exploitation of the family (19).

In this study, we exercised that, the information that Zeytinoğlu gives is true. Although almost all of the children like the school; 62.8% of them are absent from the school, and 20.6% of the children cannot go to the school since they work and these data show that working children give up the elements that are important for them. There is a strong relationship between poor educational possibilities of working children and becoming poor household individuals and household heads in the future. Education and school are among the most effective instruments in terminating the child labor. Both primary education and occupational education should become widespread. The direct and indirect costs of the education should be brought into a state that the poor families can also endure; the quality of education should be improved, and should be brought into a state providing employment in the future (2, 13, 20).

Almost half of working children expressed that they worked in repair works and made apprenticeship. This was followed by working in bakery and working as agricultural worker (Table 2). Mostly the students worked as apprentice (83.9%). The present study shows that the number of the children making repair and apprenticeship was less, that agricultural labor was more (3). The results of 2006 Child Labor Study arranged by TUİK show that in the age group of 6–17, the number of children working in economic activities decreases as a total of all sectors in the rate of 58% in the last 12 yr. The number of children working in economic activities that are totally 2270000 in 1994 regressed to 959000 in 2006. This regression largely results from the decrease in the number of children working in the agricultural sector. This regression in the last 12 yr actualized for the age group of 6–17 as 74% in agricultural sector; 30% in industrial sector; and 54% in trade sector (3, 6). However, these results are not correct for Sanliurfa. Agricultural labor is an important problem for Sanliurfa. The children working in agriculture should be kept in a separate place due to the conditions they are in. First of all, these children take place among the most disadvantaged in terms of working and living conditions, relationships with the environment, and problems of education and health. The ages of majority of these children working in the agricultural works are less than 15 and they are the children, who are not in the working age or not desired to work in the works of interest, according to ILO conventions. These children, deprived of educational possibilities, and cannot attend to school or have difficulties in attending due to working, spend the 4–7 months of the year out of the place they permanently dwell, and as a result, they are deprived of the main needs (5, 22).

The 62.2% of the children answered the question; “According to you, should the children work?” with; “No”. In this study, when asked, “What are the disadvantages of working?” 4% expressed that it reduced school success, and 29.4% told that their health is disordered. 31.3% of the children experienced tiredness due to work, and 34.5% of them experienced a backache and headache. When regarded to the studies carried out, 68.4 % of the children are satisfied with working; 31.6% not satisfied (16). In this study, the children were not satisfied while working. However, due to the poverty of the family, the child could think that he/she had to work. When the reasons for working with children were examined, most of them expressed that the money they made delivered to their families. In another study, contribution to the income of the family takes place in the first order with the rate of 34.6% (16). In all over Turkey, 41.4% of the working children work to contribute to the income of household. The children point out the low income as the cause of their working. The studies show that the main reason for child labor is poverty. Poverty is the case not to be able to reach minimum life conditions and not to be able to meet the main needs. The problem with poverty emerges, depending on problems such as income inequality, unemployment, not being able to use the resources effectively, rapid population increase, migration, unrecorded economical facilities. The children view working in street as an instrument eliminating both poverty and deprivation and exhibit an attitude making right the existing situation. Therefore, poverty normalizes the fact of working children outside and legalizes it (2, 5, 7, 8).

In this study, the rate of smoking cigarette among working children was 14.0%, the rate of using drug was 4.5%. According to WHO report, in 2008 the rate of smoking cigarette between the ages of 13–15 is 11.1% in boys; this rate is 4.4% in girls. For both genders, the common value is 8.4% (21).

In the study, carried out in Sanliurfa, the rates of smoking cigarette and using drug highly increased. This relationship was found statistically significant.

There are extremely unhealthy environments for children who spend their time away for any reason from the protection and observation of their family or society. One of the adverse effects of these environments is violence towards working children. In this study, while the rate of children exposing to violence was 18%, the rate of exposed to violence due to working was 14.7%. Children were exposed to violence by either their fathers or by the owner of workplace. Thus, every kind of behavior including violence exerted by the people regarded as authority considered normal after certain times by the child. Violence effects child development adversely, it leads to the deviations to violence. Violence causes emotional fragmentations and development of the deficient self-respect phenomenon (15, 23).

## Conclusion

Child labor is not a fact especially caused by a single reason. However, not to let the child labor turn into an intergenerational vicious circle and in terms of eliminating child labor, struggling with poverty is extremely important.

The obligation of the incorporation of children to working life is no longer a problem by developing policies struggling with unemployment, poverty, lack of education of families and traditional viewpoint, unplanned migration and unhealthy urbanization, the problems in educational system.

## Ethical considerations

Ethical issues (Including plagiarism, informed consent, misconduct, data fabrication and/or falsification, double publication and/or submission, redundancy, etc.) have been completely observed by the authors.
